# *Trachyspermum ammi* Bioactives Promote Neuroprotection by Inhibiting Acetylcholinesterase, Aβ-Oligomerization/Fibrilization, and Mitigating Oxidative Stress *In Vitro*

**DOI:** 10.3390/antiox13010009

**Published:** 2023-12-20

**Authors:** Himadri Sharma, Hyewon Yang, Niti Sharma, Seong Soo A An

**Affiliations:** Department of Bionano Technology, Gachon Bionano Research Institute, Gachon University, 1342 Seongnam-daero, Sujeong-gu, Seongnam-si 461-701, Gyeonggi-do, Republic of Korea

**Keywords:** H_2_O_2_-induced oxidative stress, Carom, anti-acetylcholine esterase activity, neuroprotection, Aβ-fibrilization, Aβ-oligomerization, Carvacrol, Thymol

## Abstract

Neurodegenerative diseases (NDs) are a large category of progressive neurological disorders with diverse clinical and pathological characteristics. Among the NDs, Alzheimer’s disease (AD) is the most widespread disease, which affects more than 400 million people globally. Oxidative stress is evident in the pathophysiology of nearly all NDs by affecting several pathways in neurodegeneration. No single drug can manage multi-faceted diseases like NDs. Therefore, an alternative therapeutic strategy is required, which can affect several pathophysiological pathways at a time. To achieve this aim, hexane and ethyl acetate extract from *Trachyspermum ammi* (Carom) were prepared, and GC/MS identified the bioactive compounds. For the cell-based assays, oxidative stress was induced in SH-SY5Y neuroblastoma cells using hydrogen peroxide to evaluate the neuroprotective potential of the Carom extracts/bioactives. The extracts/bioactives provided neuroprotection in the cells by modulating multiple pathways involved in neurodegeneration, such as alleviating oxidative stress and mitochondrial membrane potential. They were potent inhibitors of acetylcholine esterase enzymes and displayed competitive/mixed-type inhibition. Additionally, anti-Aβ_1-42_ fibrilization/oligomerization and anti-glycation activities were also analyzed. The multi-faceted neuroprotection shown via Carom/Carvacrol makes it a prospective contender in drug development for NDs.

## 1. Introduction

Neurodegenerative diseases (NDs) (neuro: brain/neuron; degenerative: deterioration) have a progressive and devastating effect on the neuronal communication network. NDs are a group of complex diseases that arise in different brain regions and affect cognition, memory, speech, body movement, balance, and much more. The central pathological developments linked with NDs are the accumulation of specific proteins, synapse dysfunction, oxidative stress, apoptosis, inflammation, and anomalies in the ubiquitin–proteasomal, autophagosomal, or lysosomal systems [1]. NDs affect millions of people worldwide, with the most common being Alzheimer’s disease (AD) and Parkinson’s disease (PD). Globally, 416 million people are affected by AD [2], and it is the fourth leading reason for disability-adjusted life-years (DALYs) lost in the elderly population [3]. As per the recent Alzheimer’s Disease Association report, the number of deaths due to AD increased by 145% between the year 2000 and 2019 [4].

Till now, there is no single drug that can manage multi-faceted diseases like AD. The present drugs are target specific viz. Donepezil, Galantamine, and Rivastigmine are acetylcholine esterase inhibitors, Aduhelm is an amyloid antibody, and Memantine acts as an NMDA receptor antagonist. Secondly, these drugs have various side effects extending from moderate dizziness to anomalies observed in brain scans [amyloid-related imaging abnormalities (ARIA)]. Hence, a multi-faceted neuroprotective strategy is necessary to improve the disease symptoms, for which the search for novel compounds with better efficacy and minimum side effects is highly desirable. In this context, the neuroprotective potential of numerous plants has been recognized [5].

Carom (*Trachyspermum ammi)* is an annual herb from the Apiaceae family. The small light-brown seed-like fruits of Carom are highly fragrant and are used as a flavoring agent in cuisines owing to their pungent and aromatic taste. Besides being used as a spice, Carom has been used in the traditional system of Indian (*Ayurveda*) and Chinese medicines in treating conditions like peptic ulcers, hypertension, hyperlipidemia, diabetes, diarrhea, arthritis, viral infections, asthma, and indigestion [6]. Carom essential oil (EO) has been known for its antibacterial, anti-fungal, insecticidal, and antioxidant properties [7]. GC-MS analysis of the EO identified Thymol and Carvacrol as the principal components, while γ-terpinene, camphene, ρ-cymene, δ-3-carene, β-pinene, myrcene, limonene, and sabinene as the minor constituents [8]. The extract and the key component provided neuroprotection and amended learning and memory in the scopolamine-induced memory deficit mice model [9]. Thymol also alleviated the symptoms of diabetic–neuropathic pain in the Streptozotocin (STZ)-induced rat model [10]. The neuroprotective role of Carvacrol has been studied in animal models of traumatic brain injury (TBI) [11], PD [12,13], and AD [14]. Additionally, the lethal dose (LD_50_) of Thymol and Carvacrol (565.7 mg/kg and 471.2 mg/kg, respectively) is higher compared to the therapeutic doses (Thymol 0.5 mg/kg, Carvacrol 1 mg/kg) which indicate efficacy and safety of these phytocompounds in improving cognitive impairments [15].

The reactive oxygen species (ROS) are highly active species with unpaired electrons which are generated as a by-product of oxygen metabolism. The elevated ROS levels have a detrimental effect on the cellular components, and as the neuronal cells are more sensitive to oxidative stress compared to others, the damage is greater. The generated ROS further accelerates mitochondrial impairment, inflammation, tau phosphorylation, neurofibrillary tangles formation, and apoptosis. Hence, shielding against oxidative stress is a significant approach in the treatment of NDs. The plants are a rich source of antioxidants and can scavenge free radicals. Consequently, the present work was planned to assess the neuroprotective properties of Carom fruit extract (hexane and ethyl acetate) and the main bioactive component, Carvacrol, on H_2_O_2_-induced oxidative stress in SH-SY5Y neuroblastoma cell lines. Moreover, additional neuroprotection mechanisms were also inspected by reviewing acetylcholine esterase (AChE) inhibition, Aβ_1-42_-oligomerization, and fibrillation inhibition activity, and anti-advanced glycation end products (AGEs) potential to explain the neuroprotective mechanism exerted by the Carom extract and bioactive compounds.

## 2. Materials and Methods

### 2.1. Chemicals

Most of the chemicals and standards for biochemical and cell-based studies were purchased from Sigma-Aldrich (St. Louis, MO, USA), namely acetyl thiocholine chloride, Acetylcholinesterase (*Electrophorus electricus*, Type VI-S), 6,6′-dinitro-3,3′-dithiodibenzoic acid, bis(3-carboxy-4-nitrophenyl) disulfide (DTNB), galantamine, bovine serum albumin (BSA), sodium azide, aminoguanidine, dextrose, 2′,7′-dichlorofluorescin diacetate (DCFDA), tetramethylrhodamine, ethyl ester (TMRE), hydrogen peroxide (H_2_O_2_), thioflavin T (ThT), gallic acid, ascorbic acid, 2,2′-azinobis-(3-ethylbenzothiazoline-6-sulfonic acid) (ABTS), 2,2-diphenyl-1-picrylhydrazyl (DPPH), 2,4,6-tripyridyl-s-triazine (TPTZ), and Folin–Ciocalteu reagent (FCR).

Thermo Fisher Scientific (Waltham, MA, USA) was the source for fetal bovine serum (FBS), kanamycin, penicillin, and phosphate-buffered saline (PBST). Dulbecco’s modified Eagle’s medium (DMEM) was supplied by Gibco (Thermo Fisher, Seoul, Republic of Korea). Carvacrol and Thymol were purchased from TCI (Chuo-ku, Tokyo, Japan). The suppliers for others: Aβ_1-42_ (AnaSpec, Fremont, CA, USA), WST-8 kit (Roche Diagnostics GmbH, Mannheim, Germany), Aβ_1–42_ for MDS (GenicBio Inc., Shanghai, China), purified anti-Aβ_1–16_ antibody (Biolegend, San Diego, CA, USA), and the horseradish peroxidase (HRP)-conjugated W_0-2_ monoclonal antibody (Peoplebio Inc., Seongnam, Republic of Korea). The HPLC-grade organic solvents were bought from Sigma-Aldrich.

### 2.2. Plant Material and Extraction

The Carom seeds (Expat Mart, Seoul, Republic of Korea) were weighed, powdered, and extracted sequentially with n-hexane and ethyl acetate. The extracted fractions were dried, weighed, and stored at 4 °C for the experiments.

### 2.3. Gas Chromatography–Mass Spectrometry (GC-MS) Method

The hexane and ethyl acetate fractions (1 μL, 1 mg/mL) were analyzed on a fused-silica capillary column (DB-5 ms UI, 30 m × 0.25 mm i.d., film thickness 0.25 μm, Agilent, Santa Clara, CA, USA) connected to GCMS-QP2020 (Shimadzu, Kyoto, Japan). The oven temperature was set (60 °C/2 min, 100 °C at 4 °C/min, 290 °C at 10 °C/min, isothermic for 10 min). The carrier gas, helium, was at a constant flow rate of 1 mL/min. The temperature was set for the injection port (280 °C), ion source (280 °C), and interface (150 °C). The ionization energy was set at 70 eV. The full scan mode (40–700 AMU) was used to obtain the mass spectra. The phytocompounds in the fractions were identified by comparing them with known compounds in the National Institute of Standards and Technology (NIST) library.

### 2.4. Determination of Total Phenolic Content

The Folin–Ciocalteu method [16] with modifications was used to evaluate the phenolic content. The extracts were incubated for 5 min at room temperature (RT) with 1 N FCR, after which a sodium carbonate solution (10%) was added. After incubating the 96-well plate for 2 h in the dark at RT, absorbance was measured at 765 nm using a multi-mode reader, Synergy-H1 BioTek, Agilent (Santa Clara, CA, USA). Calibration was performed using Gallic acid (10–200 mg/mL) as a standard, and the results were represented as a mg gallic acid equivalent (GAE)/g of the extract.

### 2.5. Determination of Total Flavonoids Content

The total flavonoids were estimated by a previously described protocol [17]. Briefly, aluminum chloride (10%), ethanol (96%), and sodium acetate (1 M) were added to the extracts. After mixing the reagents in a 96-well plate, incubation for 40 min at RT in the dark was performed, after which the absorbance at 415 nm was measured using a microplate reader (Synergy-H1 BioTek, Agilent, Santa Clara, CA, USA). Quercetin (10–100 μg/mL) was used as the standard to calculate the total flavonoids in the extracts, and the results were expressed in terms of mg quercetin equivalents per gram of sample (QE/g).

### 2.6. Determination of Antioxidant Capacity

#### 2.6.1. 2,2′-Azino-bis (3-Ethylbenzothiazoline-6-Sulfonic Acid) [ABTS] Radical Scavenging Assay

The extracts were assessed for free radical scavenging activity using a previously reported method [18]. To produce ABTS radicals, ABTS (0.7 mM) and potassium persulfate (2.45 mM) were mixed in equal ratios. The mixture was incubated at RT in the dark for 30 min. The extract and ABTS radical solution were mixed and incubated in the dark for 30 min at RT. The absorbance was monitored at 734 nm using the microplate reader (Synergy-H1 BioTek, Agilent, USA). Ascorbic acid (100 μg/mL) served as a standard. The percentage ABTS^+.^ scavenging activity was calculated using the formula:

% RSA = (AB − AE/AB) *×* 100

where AB = absorbance of the blank and AE = absorbance of the extract.

#### 2.6.2. Free Radical Scavenging using a 2,2-Diphenyl-1-picrylhydrazylhydrate (DPPH) Radical Assay

The DPPH radical scavenging activity was assessed [19] by incubating the extract with ethanolic DPPH (120 μM) in the dark at RT for 30 min, and the absorbance was read at 515 nm (Multi-mode reader, Synergy-H1 BioTek, Agilent, Santa Clara, CA, USA). Ascorbic acid (0.1–10 μg/mL) was used as a positive control. Radical scavenging activity (RSA) was calculated using the following formula:

% RSA = (AB − AE/AB) *×* 100

where AB = absorbance of the blank and AE = absorbance of the extract.

#### 2.6.3. Ferric-Reducing Antioxidant Potential (FRAP) Assay

The metal-chelating ability of the extracts was evaluated using the FRAP assay [20]. The extract was incubated with the FRAP reagent at RT for 30 min, and the absorbance was read at 593 nm (Multi-mode reader, Synergy-H1 BioTek, Agilent, USA). For the standard, FeSO_4_·7H_2_O (1 mM) was used, and the FRAP values were expressed as μM Fe^2+^/g.

### 2.7. Anti-Acetylcholinesterase Activity

The anti-AChE activity of the extracts/bioactives was measured [21] by incubating the extracts with AChE and 10 mM ATCC at 37 °C for 15 min. The absorbance was read at 412 nm (Multi-mode plate reader, Synergy-H1 BioTek, Agilent, USA) after adding a stopping reagent (DTNB). Galantamine was used as a positive control. The percent inhibition was calculated as follows:

Percent Inhibition (I%) = [(A_1_ − A_2_) − (B_1_ − B_2_)]/(A_1_ − A_2_) *×* 100

where A_1_ is the absorbance without the inhibitor; A_2_ is the negative control without the inhibitor; B_1_ is the absorbance with the inhibitor; and B_2_ is the negative control with the inhibitor. The IC_50_ values were calculated using GraphPad Prism 10.0.

### 2.8. Anti-Advanced Glycation End-Product (AGE) Activity

For the glycation reaction [22], the extract was incubated for 2 weeks at 37 °C with 100 mM and a pH 7.4 phosphate buffer [containing 50 mg/mL BSA, 0.5 M dextrose monohydrate, and 5 mM sodium azide]. Aminoguanidine served as a positive control in the assay. The fluorescence was read at Ex 370 nm/Ems 440 nm (Synergy-H1 BioTek, Agilent, Santa Clara, CA, USA), and the percent glycation inhibition was calculated as:

Inhibition (%) = [(FC − FT)/FC × 100]

where FC and FT are fluorescence intensity in the absence and presence of the sample, respectively; the IC_50_ values were calculated using GraphPad Prism 10.0.

### 2.9. Anti-Aβ_1–42_-Fibrilization Activity

The anti-Aβ_1–42_-fibrilization activity of the extracts/bioactives was monitored using a ThT assay [23]. The samples were incubated in the presence/absence of Aβ_1–42_ at 37 °C for 24 h. The samples were incubated with 100 μM ThT at 37 °C for 15 min. The fluorescence was monitored at Ex 450 nm/Ems 490 nm (Synergy-H1 BioTek, Agilent, Santa Clara, CA, USA). For the control, Phenol red (50 μM) was used. The Aβ_1–42_ aggregation inhibition was calculated as follows:

Percent Inhibition (%) = [(1 − FI/FC) × 100]

where FI and FC are the fluorescence intensity with and without the inhibitors, respectively.

### 2.10. Anti-Aβ_1–42_-Oligomerization Activity

The anti-Aβ_1–42_-oligomerization activity of the extracts/bioactives was monitored using a Multiple Detection System (MDS) as described previously [24]. Briefly, the extracts and Aβ_1–42_ were incubated at RT for different time points (0 h, 2 h, and 4 h). The samples were incubated on an anti-β-amyloid pre-coated plate for 1 h at RT. A HRP-conjugated W_0-2_ monoclonal antibody was added, and the plate was kept at RT for 30 min. Later, TMB was added, and the plate was incubated for 15 min at RT. The absorbance was read at 450 nm using a microplate reader (Victor3, PerkinElmer, CT, USA).

### 2.11. Cell Culture

SH-SY5Y human neuroblastoma cells (ATCC CRL-2266, Manassas, VA, USA) were maintained in DMEM media containing FBS (10%), kanamycin (1%), and penicillin (1%) with 5% CO_2_, and a 95% humidified atmosphere in the incubator set at 37 °C. The cells were passaged twice per week and used at 80–90% confluency.

#### 2.11.1. Cell Viability Assay

The cell viability assay was conducted as previously described [24]. In short, 1 *×* 10^4^ cells/well were seeded in 96-well plates and incubated for 24 h with various concentrations of extracts/bioactives with the final volume of 100 µL/well. The extracts/bioactives were removed from the cells and then washed twice with 1X PBS. The cells were incubated in a fresh medium containing a 10% WST-8 reagent for 2 h. The absorbance was read at 450 nm using a multi-plate reader (Synergy-H1 BioTek, Agilent, USA). The percent cytotoxicity was calculated as:

Cytotoxicity % = (A_C_ − A_T_)/(A_C_) *×* 100

where A_C_ = absorbance of the control cells and A_T_ = absorbance of the treated cells.

#### 2.11.2. Neuroprotective Activity Assay

The neuroprotective effect of extracts/bioactives on H_2_O_2_-induced oxidative stress in SH-SY5Y was evaluated [24]. The cells were seeded 1 *×* 10^4^ cells/well in a 96-well plate and incubated for 24 h with various concentrations of extracts/bioactives with a final volume of 100 µL/well. The extracts/bioactives were drawn out from the cells and incubated with 100 μM H_2_O_2_ for 6 h. The cell viability was evaluated using a WST-8 reagent (as mentioned in Section 2.11.1).

#### 2.11.3. Measurement of Intracellular Reactive Oxygen Species

The ROS was measured using H_2_DCFDA dye as previously described [24]. H_2_DCFDA is a nonpolar dye that easily diffuses into cells and is hydrolyzed to 2′,7′-dichloro dihydrofluorescin (DCFH) via intracellular esterase. DCFH is oxidized to 2′,7′-dichlorofluorescein (DCF), a highly fluorescent compound, due to ROS production in the cells. Thus, the fluorescence intensity is proportional to the amount of hydrogen peroxide/ROS produced by the cells. The 1 *×* 10^4^ cells/well were plated and incubated in a 96-well plate for 24 h with various concentrations of extracts/bioactives with a final volume of 100 µL/well. The extracts/bioactives were drawn out from the cells and incubated with 100 μM H_2_O_2_ for 4 h. The cells were then incubated with 25 μM H_2_DCFDA dye in the dark for another 2 h at 37 °C. The fluorescence intensity (Ex 495 nm/Ems 520 nm) was monitored using a multi-mode microplate reader (Synergy-H1 BioTek, Agilent, USA). The ROS was calculated as a percentage of the untreated control cells (100%) in triplicate measurements.

#### 2.11.4. Mitochondrial Membrane Potential (ΔΨm) Assay

TMRE staining was used to evaluate the mitochondrial membrane potential [25]. The cells were seeded (1 *×* 10^4^ cells/well) in a 96-well plate and pre-treated with the extracts/bioactives for 12 h. After removing extracts/bioactives from the wells, the cells were incubated with H_2_O_2_ (200 μM) for 2 h. After the incubation, H_2_O_2_ was removed, and the cells were treated with 1 μM TMRE for 1 h at 37 °C. The fluorescence intensity (Ex 549 nm/Ems 575 nm) was monitored using a multi-mode microplate reader (Synergy-H1 BioTek, Agilent, USA). The ΔΨm was expressed as a percentage of the untreated control cells (100%).

### 2.12. Statistical Analysis

Statistical investigation was performed using a one-way analysis of variance (ANOVA) followed by Dunnett’s post hoc test. Data were reported as the mean *±* SD of three experiments. Data were considered to be significant ####/**** *p* < 0.0001; ###/*** *p* < 0.001; ##/** *p* < 0.01; and #/* *p* < 0.05. The symbol # indicates significance compared to the H_2_O_2_ control, whereas * indicates significance compared to the untreated control. The IC_50_ values were calculated using non-linear regression. The Michaelis–Menten plot using a non-linear fit via GraphPad Prism 10.0 was used to calculate Vmax and Km values. Lineweaver–Burk plots were designed via linear regression on GraphPad Prism 10.0.

## 3. Results and Discussion

### 3.1. Phytochemical Evaluation and Antioxidant Ability of Carom Extract

The plant’s secondary metabolites (phenols and flavonoids) have vital roles in plant growth and communication besides having versatile medicinal benefits for human health. Lately, the contribution of total phenolic (TPC) and flavonoids (TFC) in promoting antioxidant activity has been highlighted [26]. In the present study, colorimetric assays were carried out to estimate TPC and TFC in the extracts. The TPC in Carom-H (Hexane) and Carom-EA (Ethyl acetate) extracts was assessed as 11.70 ± 0.32 mg GAE/g and 5.61 ± 0.04 mg GAE/g, respectively. The TFC in the Carom-H (9.79 ± 0.06 mg QE/g) was lower than the Carom-EA (14.3 ± 1.09 mg QE/g).

Various assays (DPPH, ABTS, and FRAP) were conducted to estimate the antioxidant potential of the extracts. The percent radical scavenging activity using the DPPH assay was 46.01 ± 1.07% and 32.58 ± 0.89% for Carom-H and Carom-EA, respectively. A similar trend was observed in the radical scavenging ABTS assay with 55.89 ± 0.45% (Carom-H) and 40.15 ± 0.34% (Carom-EA). The FRAP assay measures the reduction in Fe^3+^ to Fe^2+^ in the presence of antioxidants. Similar FRAP values were obtained for Carom-H (1.56 ± 0.06 mM Fe^2+^/g) and Carom-EA (1.25 ± 0.03 mM Fe^2+^/g). A positive correlation between phenolic content and the antioxidant potential of Carom-H was observed in this study.

TPC values varied from 16.52 to 43.2 mg GAE/g and TFC from 3.89 to 8.03 mg QE/g in different varieties of methanolic Carom extracts [27]. The antioxidant activities quantified in the same study displayed 6.23–10.31 mM Fe^2+^ in the FRAP assay and 65–80% radical scavenging activity in the DPPH assay [28]. The FRAP values varied from 0.67, 0.71, and 2.27 mM Fe^2+^/L for aqueous, methanolic, and acetone extract, respectively [28]. Thus, the existence of phenolic monoterpenes (Thymol and Carvacrol) in the extracts could be the reason for antioxidant activity [29]. These compounds possess good reducing potential and free radical scavenging capacity besides preventing hydroperoxydiene formation in the initial steps of lipid degradation [30].

### 3.2. GC–MS Analysis of Carom Extract

To identify the phytochemicals in dried Carom fruit extracts (Hexane and Ethyl acetate), GC–MS analysis was carried out. The chromatogram identified Phenol, 2-methyl-5-(1-methylethyl)-(Syn. Carvacrol) as the major peak in both hexane (87.17%) and ethyl acetate (80.15%) extracts at 11.5 min (Supplementary Figure S1) while the other compounds were present in less than 2% (hexane) and 4% (ethyl acetate) extracts.

**Figure 1:**
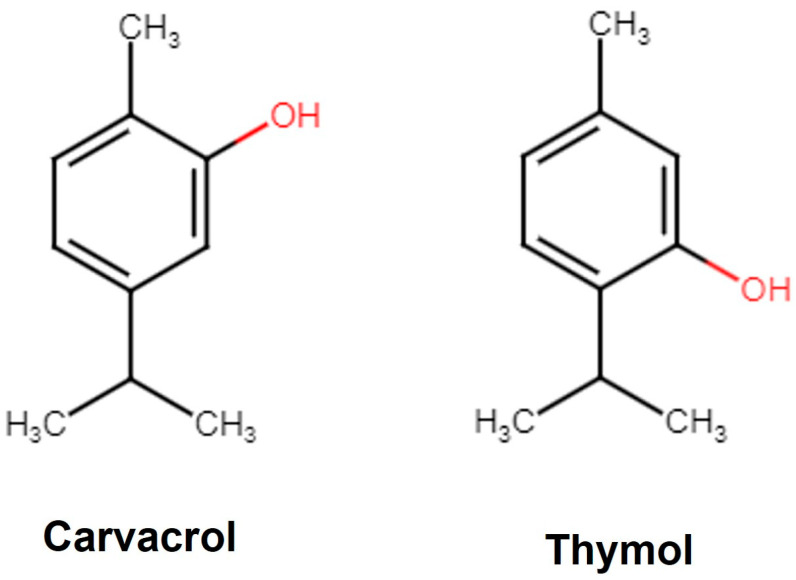
Structure of Carvacrol and Thymol. Carvacrol is the phenol isomer of Thymol.

Carvacrol is a phenolic monoterpene derivative of Cymene (Figure 1) with a characteristic pungent odor and is a component of herbs like thyme, sage, and oregano. In plants, Carvacrol is synthesized by the mevalonate pathway, where the mevalonic acid is converted to γ-terpinene and forms Carvacrol via *p*-Cymene hydroxylation in the following steps [31]. Carvacrol is reported to exhibit anticancer [32], antidiabetic [33], anti-obesity [34], anti-inflammatory [35], antimicrobial [36], anti-asthmatic [37], and antiaging [38] properties. Carvacrol has a drug-like favorable pharmacokinetic and physiochemical profile (MW 150.22; logarithm of partition coefficient [log *P* 3.81]; hydrogen bond acceptor 1 [HBA 1]; hydrogen bond donor 1 [HBD 1]) including gastrointestinal (GI) absorption, blood–brain barrier (BBB) penetration [39], and inhibition of the Cytochrome (CYP_450_) complex [40]. The Food and Drug Administration (FDA) and the European Council have approved Carvacrol as a food flavoring agent [41]. Thymol [2-isopropyl-5-methylphenol] is an isomer of Carvacrol (Figure 1) with several therapeutic properties like anti-inflammatory, anti-fungal, and antioxidant [42]. It is also recognized as a safe food flavoring agent by the FDA [43].

### 3.3. In Vitro Anti-Acetylcholinesterase Activity

**Figure 2:**
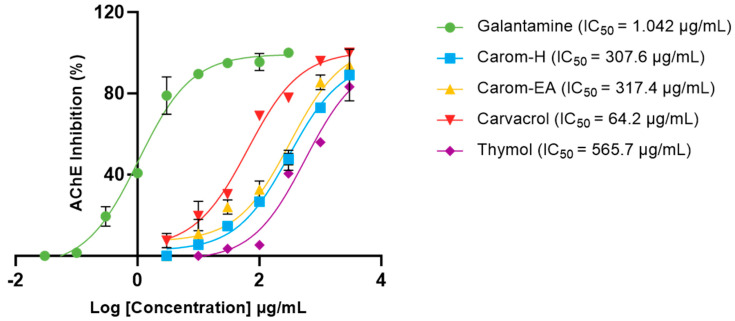
IC_50_ curves of Carvacrol, Thymol, Carom-H, and Carom-EA extracts against Acetylcholinesterase (AChE) from *Electrophorus*. Galantamine was used as a standard inhibitor control. The IC_50_ values were calculated using GraphPad Prism 10.0.

Acetylcholinesterase (AChE; E.C.3.1.1.7) is a cholinergic enzyme generally present at the postsynaptic neuromuscular junctions. It hydrolyses acetylcholine (ACh), an important neurotransmitter that plays an essential role in the cholinergic signaling pathway. This hydrolysis brings cholinergic neurons to return to the resting state [44]. In AD, the ACh level declines in the synaptic junction; hence, inhibition of AChE is desirable to maintain normal ACh levels. Therefore, the extracts and Carvacrol were examined for anti-AChE activity, and the IC_50_ values (half-maximal inhibitory concentration) were calculated using Galantamine hydrobromide as the inhibitor control. The IC_50_ values of extracts/bioactives were 307.6 μg/mL (Carom-H), 317.4 μg/mL (Carom-EA), and 64.21 μg/mL (Carvacrol) (Figure 2). We have also calculated IC50 of Thymol (565.7 μg/mL), an isomer of Carvacrol (Figure 1). The IC_50_ value of Galantamine was 1.042 μg/mL, similar to an earlier value of 1.78 μg/mL [45]. The IC_50_ value for Carvacrol obtained in our experiment was similar to previously obtained values of 288.26 μM (43.29 μg/mL) [46] and 91.7 μg/mL [29]. However, a recent study reported a lower IC_50_ value (3.8 μg/mL) for Carvacrol and Galantamine (0.6 μg/mL) [47].

The mode of inhibition of the Carom extracts, Carvacrol, and Thymol was analyzed using the Lineweaver–Burk plot. The Vmax (the maximum reaction rate when the enzyme is saturated with substrate), Km (the substrate concentration that enables the enzyme to reach half Vmax), and inhibition pattern were summarized in Table 1.

**Figure 3:**
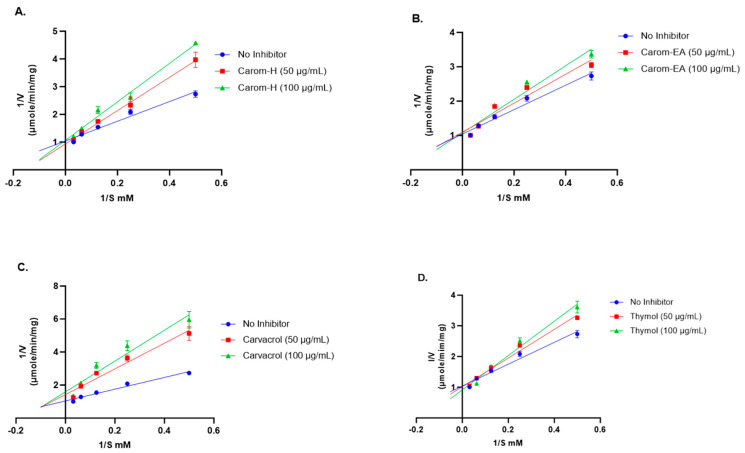
Lineweaver-Burk plots of Acetylcholinesterase in the presence of 50 μg/mL and 100 μg/mL of Carom-H (**A**), Carom-EA (**B**), Carvacrol (**C**), and Thymol (**D**). The graphs were plotted using GraphPad Prism 10.0. Abbreviations: V: velocity of enzyme-catalyzed reaction; S: substrate.

Competitive inhibition was observed via the Carom extracts, Carvacrol, and Thymol (Figure 3). From the results obtained (Table 1), the value of Vmax with extracts/Carvacrol did not deviate much from the Vmax value of the no-inhibitor (1.096 μmol/min/mg). On the other hand, the Km value increased in the presence of extracts, Carvacrol, and Thymol as compared to no-inhibitor (5.02 mM). This trend indicates a competitive inhibition where the substrate molecule and inhibitor compete for binding with the enzyme’s active site. In the presence of an inhibitor, amplified Km decreases the enzyme’s affinity for the substrate; hence, a greater substrate concentration is needed to attain Vmax. However, in the Lineweaver–Burk plot, Carvacrol exhibited mixed inhibition (combination of competitive and uncompetitive inhibition) (Figure 3), where it can bind to both a free enzyme (E) as well as a substrate-bound enzyme (ES). The higher Km value obtained in the presence of Carvacrol indicates that it occupies the enzyme active site for a more extended period, reducing the enzyme’s affinity for the substrate, which makes it the best inhibitor.

The IC_50_ values obtained were similar for the hexane and ethyl acetate extracts of Carom, suggesting the involvement of Carvacrol, the major component, in anti-AChE activity. The IC_50_ of Carvacrol was better than the extracts because it was a single purified compound. However, a significant difference was observed in the IC_50_ values of Carvacrol and Thymol, even though they were isomers. Previously, Carvacrol exhibited 10 times stronger anti-AChE activity compared to its isomer, Thymol [48,49]. This indicates the importance of the hydroxyl group at the para-position (as in Carvacrol) for effective binding to the AChE active site. The molecular docking studies showed that Carvacrol binds to the active site of human AChE through hydrogen bonding between the para-hydroxyl group and Asp_74_ side chain besides the π–π interaction with the side chain of Tyr_341_ [50]. In yet another in silico study, Carvacrol showed a docking score of −6.060 Kcal/mol and −6.986 Kcal/mol against AChE in Standard Precision (SP) and Extra Precision (XP) Glide docking, respectively. Additionally, Carvacrol interacted with Tyr_334_, Phe_330_, Trp_432_, Tyr_442_, Trp_84_, and His_440_ at the enzyme’s active site [50]. On the other hand, Thymol interacted with the active site’s Ser_203_ and His_447_ with a binding free energy (Δ*G*_bind_) of −18.49 kcal/mol [51].

### 3.4. In Vitro Anti-Glycation Potential of Carom

We monitored the *in vitro* anti-glycation potential of the Carom extracts, Carvacrol, and Thymol using a BSA-AGE fluorescence assay. At a concentration of 1 mg/mL, a weak anti-glycation effect via Carom-H (19.14 ± 0.75%), Carom-EA (21.22 ± 0.99%), Carvacrol (20.76 ± 1.47%), and Thymol (12.92 ± 0.6%) was seen. AGEs are known to be involved in the pathogenesis of several diseases. Plant extracts exert anti-glycation potential by reducing the interaction of proteins and sugars by interfering with the lysine residues in the protein. A previous study reported 13.53% glycation inhibition via the aqueous extract [52], while the hydro-ethanolic extract exerted 80% inhibition at 1 mg/mL [53]. On the contrary, Carvacrol and Thymol displayed a frail anti-AGE activity compared to the methanolic plant extract [54]. Interestingly, the synergistic action of the Thymol and p-Cymene (2:1) mixture effectively reduced the glycation as compared to Thymol alone (5 mg/mL) [55]. The antiglycation activity might have resulted either from its antioxidant nature and/or by masking the sugar-binding site in the protein [55]. However, we observed a feeble anti-glycation effect of Carom extracts and pure compounds at 1 mg/mL, indicating that a higher concentration might be effective in exerting the potent effect.

### 3.5. Effect of Carom on Aβ-Fibrilization and Oligomerization

**Figure 4:**
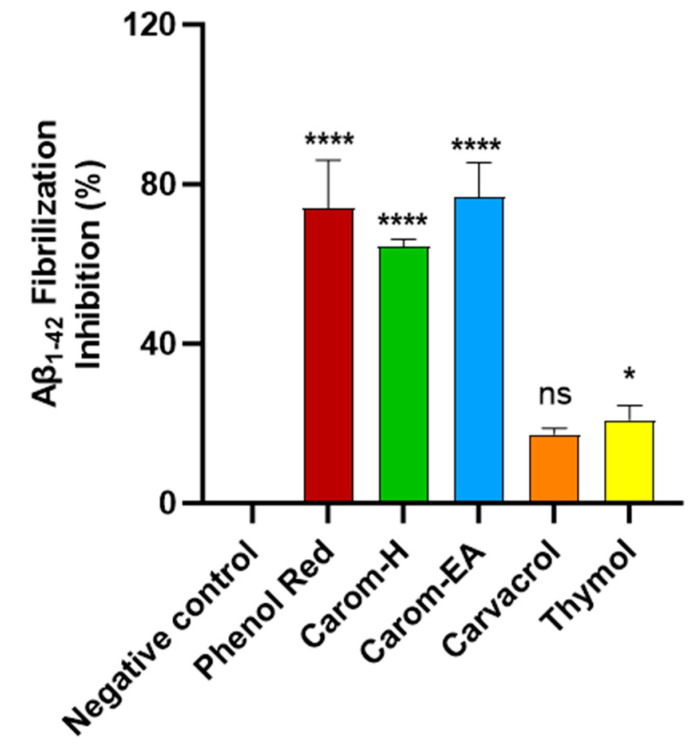
Aβ-fibrilization inhibition in the presence of Carom extracts, Carvacrol, and Thymol at 500 μg/mL. Phenol Red (50 μM) served as a positive control. The values were expressed as the mean ± SD (*n* = 3). A significant difference (* *p* < 0.05) and (**** *p* < 0.0001) using one-way ANOVA followed by Dunnett’s post hoc was observed in the reduction in oligomerization vs. the negative control (buffer + Aβ). Statistically non-significant data were represented as ns.

Protein aggregation to form amyloid fibrils is a general characteristic of diseases like AD, PD, and type 2 diabetes. Thioflavin T (ThT) is the commonly used probe to perceive *in vitro* amyloid fibrilization. The fluorescence of ThT increases upon binding to amyloid fibrils due to the rotational immobilization of the C–C bond linking the benzothiazole and aniline rings [56]. We screened the samples at 500 μg/mL where Carom-EA exerted the best Aβ-fibrilization inhibition (76.78 ± 8.63%; **** *p* < 0.0001), followed by Carom-H (64.38 ± 1.74%; **** *p* < 0.0001), Carvacrol (17.33 ± 1.55%; ns), and Thymol (20.91 ± 3.61%; * *p* < 0.05) (Figure 4). The positive control (Phenol red) exhibited 76.42 ± 7.98% at 50 μM, similar to a previously reported value [23].

In an earlier report, Carom seed aqueous extract exhibited a 30.39% reduction in β-amyloid aggregation in a ThT assay [52]. The π-stacking between Aβ aromatic side chains (especially diphenylalanine at the 19–20 position) is accountable for amyloid aggregation. The aromatic rings in the compounds might interact with diphenylalanine’s π stacking to exert anti-amyloidogenic activity [57]. The aromatic compounds are known to compete with polypeptide monomers for interaction with the extending fibrils, leading to fibrillization inhibition. Additionally, the OH groups probably perform an auxiliary role for effective interaction with β-sheet fibrils [58]. Therefore, it is reasoned that aromatic compounds in the extract stabilize the protein β structure through π-stacking or hydrophobic interaction [59]. However, groups other than the phenolic group are required for interactions with the protein for potent activity [60]. We suggest that in the present study, the mild fibrilization inhibition exhibited by Carvacrol (17.33%) and Thymol (20.91%) is due to the interaction of the phenolic ring with the growing Aβ. The lower inhibitory activity of pure compounds compared to the extract signifies the synergistic or additive involvement of other phytocompounds in the extracts for the anti-amyloidogenic property. Among the compounds identified by GC-MS (Figure S1), cis-9-Octadecenoic acid and carbamate derivative have been reported to modulate Aβ aggregation [61,62] and also possess antioxidant and anti-inflammatory activities [63]. However, the literature on the effect of other compounds on Aβ aggregation is lacking. Therefore, it can be speculated that their presence exerted a synergist effect to improve anti-fibrilization activity in the extracts compared to the pure compounds.

**Figure 5:**
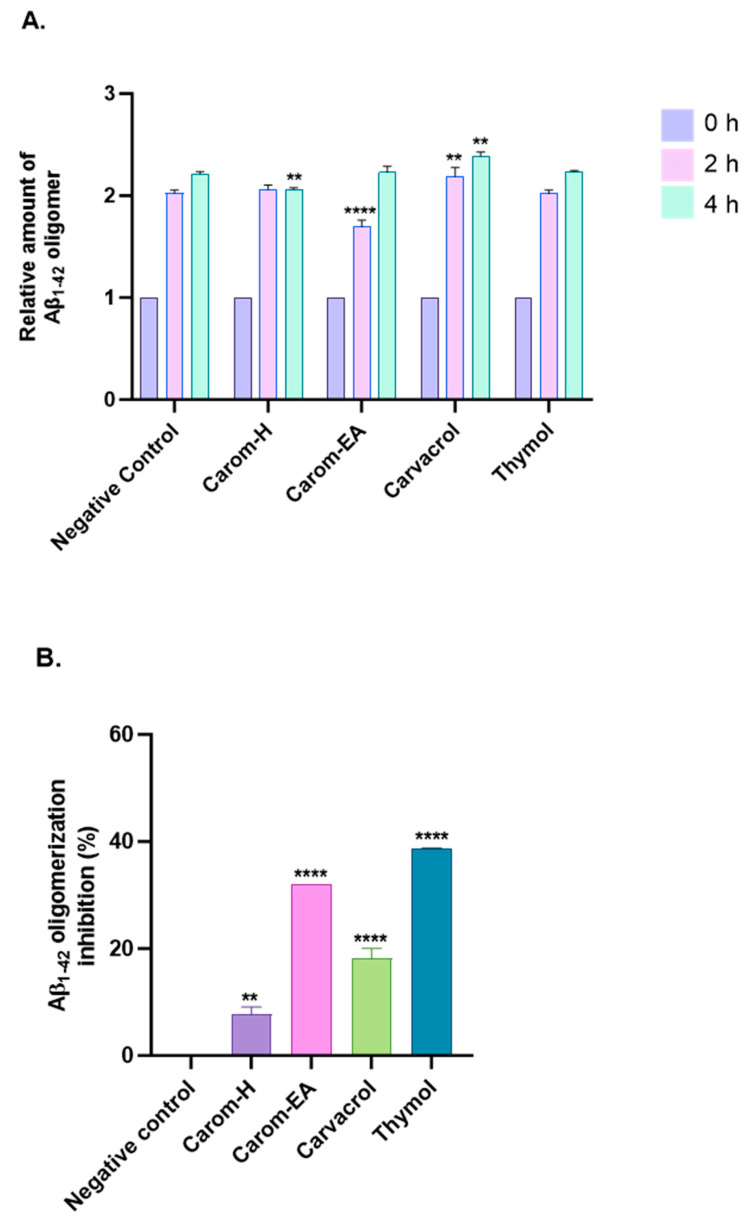
Aβ-oligomerization inhibition in the presence of Carom extracts, Carvacrol, and Thymol. (**A**) The relative amount of Aβ oligomers at 0, 2, and 4 h. (**B**) value of Aβ oligomerization inhibition. All data are expressed as mean ± SEM (n = 3). A significant difference ** (*p* < 0.01), and **** (*p* < 0.0001) using the two-way ANOVA (**A**) and one-way ANOVA (**B**) followed by Dunnett’s post hoc was observed in the percent oligomerization reduction vs. the negative control (Buffer + Aβ).

Aβ oligomerization inhibition via Carom extract and the bioactives was investigated using MDS. To evaluate the anti-Aβ oligomerization potential, the extracts/bioactives were treated with Aβ_1–42_ for different time intervals (Figure 5A and Supplementary Figure S2). The results were evaluated based on the 0 h value, which is why all sample signals were set at 1.0. The oligomerization reduction at 2 h was statistically significant in Carom-EA (**** *p* < 0.0001) and Carvacrol (** *p* < 0.01). Though a reduction in oligomerization was observed with Thymol, the *p*-value was insignificant. After 4 h of incubation, a statistically significant oligomerization reduction (** *p* < 0.01) was observed via Carom-H and Carvacrol. The compounds may inhibit Aβ oligomerization at different times due to structural differences affecting their interaction with the protein (Aβ). Moreover, degradation of compounds may occur with time, which this assay cannot observe.

The percentage inhibition of oligomerization is displayed in Figure 5B. Carom-EA and Carvacrol significantly inhibited Aβ oligomerization: 32.03% (**** *p* < 0.0001) and 18.12 ± 1.92% (**** *p* < 0.0001), respectively. However, Carom-H had a nominal effect (6.67 ± 0.02%; ** *p* < 0.01) on Aβ oligomerization inhibition (Figure 5B). The lower inhibition displayed via Carom-H could be due to higher concentrations of other metabolites in Carom-EA, which might synergistically enhance inhibition in Carom-EA. Interestingly, Thymol was more effective (38.67 ± 0.05%; **** *p* < 0.0001) than its isomer, Carvacrol, indicating the importance of the meta-hydroxyl group in oligomerization inhibition.

Amyloid aggregation is a crucial event in the pathogenesis of many neurodegenerative diseases. Therefore, inhibiting amyloid aggregation is an effective approach to treating/preventing such diseases. A recent study [64] investigated the role of central (ϕ_1_), C-terminal hydrophobic (ϕ_2_), and C-terminal end (ϕ_3_) of Aβ_42_ in the aggregation process. It was hypothesized that the ϕ_1_ domain drives Aβ_42_ amyloid aggregation, so compounds interacting with this domain suppress amyloid aggregation. The phenolic compounds are known to prevent Aβ oligomerization through the direct interaction of the phenolic hydroxyl group with the protein’s histidine or lysine side chain [65]. The compounds that inhibit Aβ assembly are classified into three categories: Class I (inhibit oligomerization but no effect on fibrilization), Class II (inhibit both), and Class III (inhibit fibrilization and no effect on oligomerization) [66]. According to the above classification, our compounds are Class II inhibitors, which stabilize Aβ conformations that do not facilitate either oligomers or fibril formation. Thymol’s better activity than Carvacrol indicates the advantage of the meta-substituted hydroxyl group in enhancing activity, as reported previously [67].

### 3.6. Non-Toxic Effect of Carom in SH-SY5Y Cells

**Figure 6:**
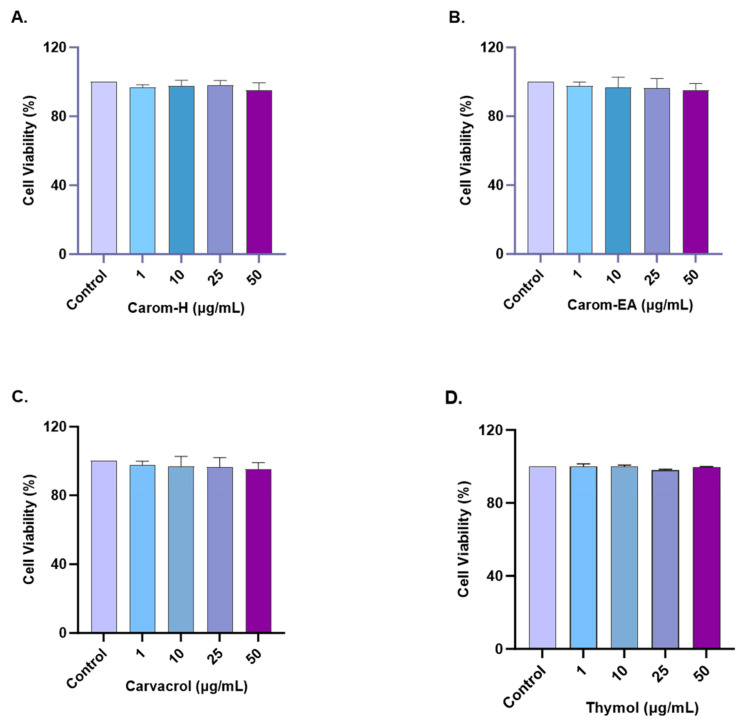
Cytotoxicity assay of Carom-H (**A**), Carom-EA (**B**), Carvacrol (**C**), and Thymol (**D**) on the SH-SY5Y cells. The cells were treated with varying concentrations (1, 10, 25, and 50 μg/mL) of extract/bioactive for 24 h. The cell viability was calculated as the percentage of the control group (100%). The data were expressed as mean ± SD (n = 3). No significant difference was observed using a one-way ANOVA followed by Dunnett’s post hoc in the % cell viability vs. the control group (no treatment).

The cytotoxic effect of Carom on SH-SY5Y cells was monitored by treating cells with different concentrations (1, 10, 25, and 50 μg/mL) of the extracts and pure compounds for 24 h. The cell viability was measured using WST-8 dye. No statistically significant cytotoxicity was examined up to 50 μg/mL for both extracts and the pure compounds (Figure 6 and Supplementary Figure S3). Our results are supported by the previous findings where Carvacrol (200 μM) and Thymol (100 μM) produced no cytotoxicity in PC12 cells [68]. Carvacrol was non-cytotoxic, up to 100 mg/L in rat neurons and N2a neuroblastoma cells [69] and up to 333 μmol/L in SH-SY5Y cells [70]. Based on our results, further experiments were conducted up to 50 μg/mL concentrations of extracts/pure compounds.

### 3.7. Neuroprotective Effect of Carom against H_2_O_2_-Induced Oxidative Stress in SH-SY5Y Cells

**Figure 7:**
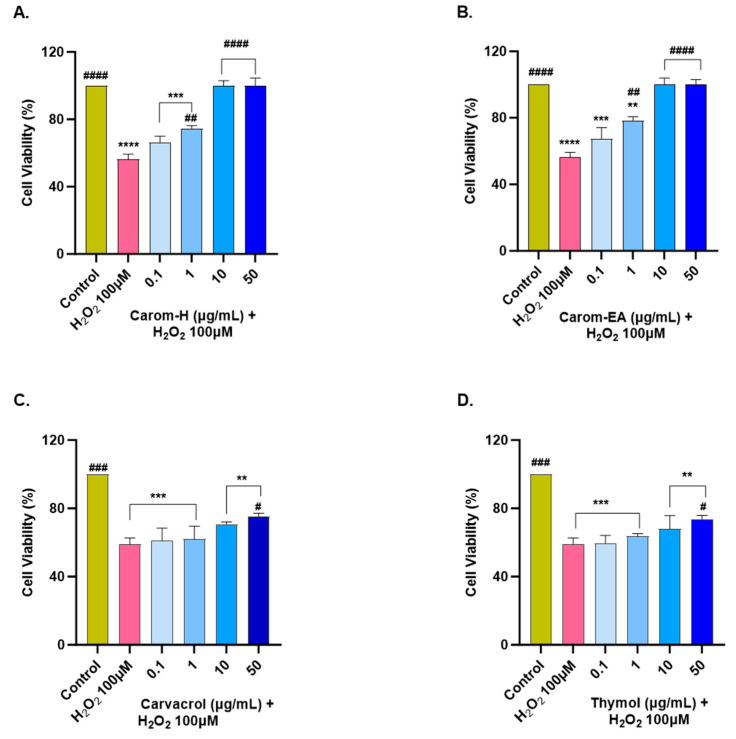
Neuroprotective effect of Carom-H (**A**), Carom-EA (**B**), Carvacrol (**C**), and Thymol (**D**) in H_2_O_2_-induced oxidative stress in neuroblastoma SH-SY5Y cells. The SH-SY5Y cells were pre-treated with various concentrations of the extract/bioactives (0.1, 1, 10, and 50 μg/mL) for 12 h followed by 6 h of H_2_O_2_ (100 μM) treatment. The data were expressed as mean ± SD (n = 3). The results were displayed in terms of % cell viability vs. the control cells. A significant difference ^#^ (*p* < 0.05), **^/##^ (*p* < 0.01), ***^/###^ (*p* < 0.001), and ****^/####^ (*p* < 0.0001) using one-way ANOVA followed by Dunnett’s test was observed in the % cell viability vs. untreated cells (*) and H_2_O_2_ treated cells (#).

The extracts and pure compounds were assessed for neuroprotective potential in H_2_O_2_-induced oxidative stress in the SH-SY5Y cells. The concentration of H_2_O_2_ was optimized as 100 μM (that resulted in approx. 50% cell viability after 6 h treatment). The cells were pre-incubated with the extracts/bioactives at different concentrations (0.1, 1, 10, and 50 μg/mL) for 12 h, after which oxidative stress was induced by incubating the cells with 100 μM H_2_O_2_ for 6 h. A similar dose-dependent neuroprotection was seen in both extracts from 0.1 μg/mL to 10 μg/mL, after which the response was constant (Figure 7 and Supplementary Figure S4). The results were non-significant at the lowest concentration (0.1 μg/mL) but displayed significant neuroprotection (74.6 ± 1.61%, ^##^ *p* < 0.01) in Carom-H, and (78.31 ± 2.41%, ^##^ *p* < 0.01) in Carom-EA, at 1 μg/mL. The neuroprotective effect was more significant at 10 and 50 μg/mL (100%, ^####^ *p* < 0.0001).

From the above results, both the extracts exerted the best neuroprotection at 10 μg/mL, after which the effect remains almost constant. A similar neuroprotective trend exerted by both extracts could be due to the main bioactive compound, Carvacrol. On the other hand, Carvacrol and Thymol presented statistically significant neuroprotection (^#^ *p* < 0.05) at 50 μg/mL. The reason for the better neuroprotective effect of the extracts compared to the pure compounds could be the synergetic/additive effect of other phytocompounds present in the extract. Our results are supported by a previous finding where 10–50 μM of Carvacrol and Thymol significantly protected PC12 cells against Aβ_25-35_-induced oxidative stress [68]. Thus, the neuroprotective action of Carom/bioactives might be due to the attenuation of oxidative stress. The better neuroprotection by extracts compared to the pure compounds could be the result of synergistic/additive action of other minor phytocompounds in the extracts. Among the identified phyto-compounds by GC-MS (Figure S1), *γ*-terpinene [71], vinyltriphenylphosphonium bromide (VTPB) [72], and cis-9-Octadecenoic acid [63] displayed potent antioxidant activity in various *in vitro* and *in vivo* conditions, which might be responsible for better neuroprotection by the extracts.

### 3.8. Alleviation of ROS Levels in SH-SY5Y Cells via Carom

Oxidative stress is generated due to the improper functioning of the antioxidant system and is a key modulator in aging and neurodegeneration. The ROS generation has adverse effects on the biomolecules, especially the neurons more prone to oxidative damage. Therefore, to study the protective effect of Carom extracts, Carvacrol, and Thymol, the SH-SY5Y cells were pre-incubated for 12 h with different concentrations of the extract/pure compounds followed by 4 h of H_2_O_2_ (100 μM) exposure. The ROS generation was monitored using H_2_DCFDA dye, which becomes oxidized in the presence of ROS to DCF, and the fluorescence was monitored at Ex 495 nm/Ems 520 nm.

**Figure 8:**
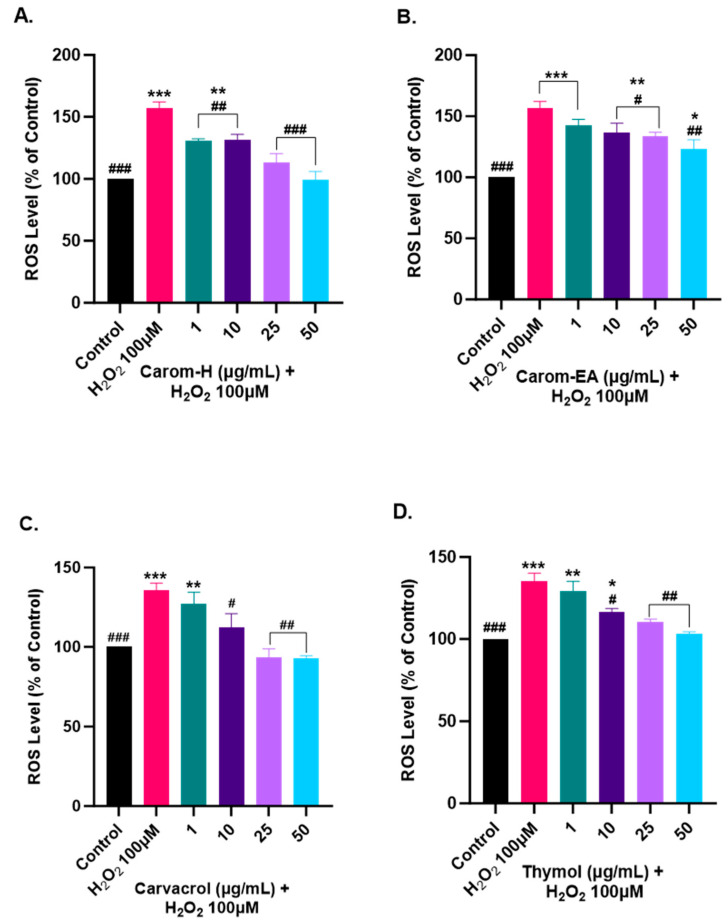
Effect of Carom-H (**A**), Carom-EA (**B**), Carvacrol (**C**), and Thymol (**D**) on H_2_O_2_-induced ROS production in SH-SY5Y cells. The SH-SY5Y cells were pre-incubated for 12 h with varying concentrations (1, 10, 25, and 50 μg/mL) of the extracts/bioactives followed by 4 h H_2_O_2_ (100 μM) exposure. The data were expressed as mean ± SD (n = 3). The results were displayed in terms of % ROS level vs. the control cells (untreated cells). The data analysis was performed usin a one-way ANOVA followed by Dunnett’s test. A significant difference *^/#^ (*p* < 0.05), **^/##^ (*p* < 0.01), and ***^/###^ (*p* < 0.001), was observed in the % ROS vs. untreated cells (*) and H_2_O_2_ treated cells (#).

Oxidative stress occurs when the cellular defense system cannot compensate for ROS production. The bioactive components are usually good antioxidants that help quench the ROS and maintain homeostasis. In our experiment, we used H_2_O_2_ to induce ROS in the cells as it can diffuse freely through cellular membranes due to its solubility in lipid and aqueous environments, affecting key cellular activities (growth, proliferation, and differentiation). We observed ~150% for the ROS level in H_2_O_2_ alone treated cells, while a dose-dependent decrease in the ROS level was observed in the cells pre-treated with the extracts/pure compounds. Carom-H is slightly more effective compared to Carom-EA (Figure 8 and Supplementary Figure S5). A statistically significant (130.48 ± 1.71%; ## *p* < 0.01) effect in reducing ROS was observed even at the lowest tested concentration (1 μg/mL) of Carom-H and almost complete alleviation of ROS level at 25 μg/mL (113.02 ± 7.41%; ### *p* < 0.001) and 50 μg/mL (99.12 ± 6.78%; #### *p* < 0.0001). On the other hand, with Carom-EA pre-treatment the ROS levels were 142.78 ± 4.63% (statistically non-significant) at 1 μg/mL, 136.77 ± 6.07% (# *p* < 0.05) at 10 μg/mL, 133.99 ± 2.39% (# *p* < 0.05) at 25 μg/mL, and 123.04 ± 7.68% (## *p* < 0.01) at 50 μg/mL. Both Carvacrol and Thymol displayed similar neuroprotective trends at 25 and 50 μg/mL (### *p* < 0.001), but Thymol displayed a better significant effect (## *p* < 0.01) than Carvacrol (# *p* < 0.05) at 1 μg/mL. The possible mechanism of a dose-dependent decrease in ROS levels signifies the extracts’ antioxidant nature, which activates the endogenous defense systems either by scavenging the free radicals or indirectly shielding them from oxidative stress [73]. The effective ROS alleviation by Carom-H could be related to better antioxidant activity compared to Carom-EA.

H_2_O_2_ and superoxide (O_2_^•−^) are the main ROS involved in the signaling pathways. The exogenous H_2_O_2_ is lethal to cells as it can easily be transformed into toxic ROS through the Fenton reaction. Additionally, the generation of hydroxyl radicals is enhanced in phosphate buffers in the Fenton reaction [74]. Oxidative stress occurs when ROS burdens the endogenous antioxidant defense and eventually results in various pathological conditions. Plants are a rich source of natural antioxidants that protect against the harmful effects of free radicals by strengthening the endogenous antioxidant defense and restoring the ideal equilibrium by counteracting free radicals [75]. Our results showed that pre-treatment of extracts/pure compounds provided protection against H_2_O_2_-induced oxidative stress in the cells via the enhancement of both non-enzymatic (GSH) and enzymatic (SOD, CAT, and GPX) endogenous antioxidant systems, which eventually protected the cells by mitigating the oxidative stress.

Alcoholic Carom extract has been reported to reduce ROS, boost antioxidant defense, and prevent apoptosis in PC12 cells [76]. In addition, extract and Thymol supplementation reduced oxidative stress, promoted neurogenesis, and reduced Aβ deposition in scopolamine-induced AD mouse model [9]. Thymol and Carvacrol are potential antioxidants that significantly reduce oxidative stress and ROS production *in vitro* [77]. Moreover, Thymol improves symptoms of neuropathic pain by reducing oxidative stress and cytokine release [10].

### 3.9. Protective Effect of Carom Extract on Mitochondrial Membrane Potential of SH-SY5Y Cells

ROS is produced in the mitochondria through electron transport, further stimulating proinflammatory cytokine production. Mitochondrial membrane potential (∆Ψm) is affected by oxidative damage to the cell, which disturbs membrane permeability by releasing Cytochrome C and/or pro-apoptotic factors in the cytoplasm. Hence, a decrease in MMP is viewed as a primary marker in events such as apoptosis and NDs.

**Figure 9:**
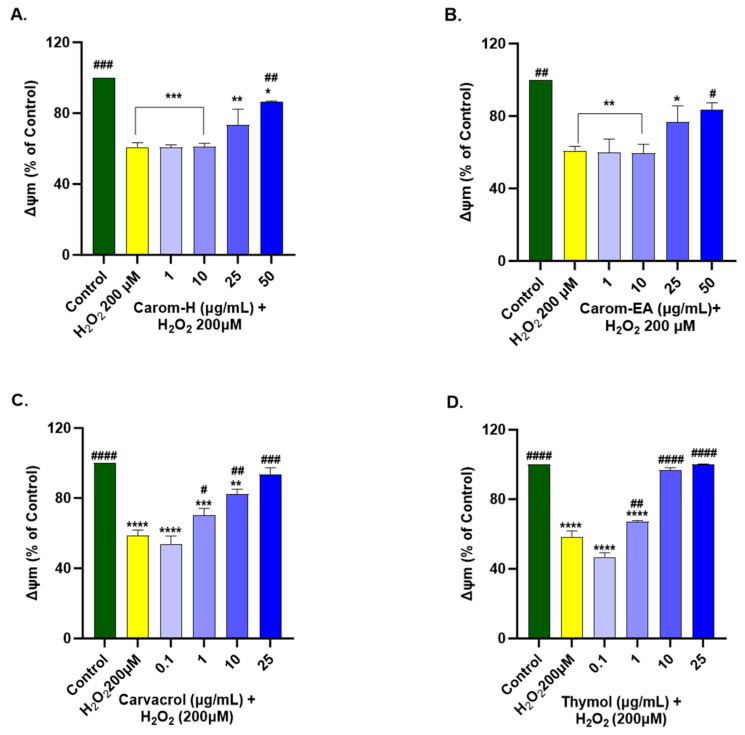
Mitochondrial membrane potential in SH-SY5Y cells exposed to 200 μM H_2_O_2_ for 2 h after 12 h pre-treatment with Carom-H (**A**), Carom-EA (**B**) extracts at 1, 10, 25, and 50 μg/mL and Carvacrol (**C**), and Thymol (**D**) at 0.1, 1, 10, and 25 μg/mL. The results were expressed as mean ± SD (n = 3) in terms of % ∆Ψm vs. the control cells (untreated cells). The data analysis was performed using the one-way ANOVA followed by Dunnett’s test. A significant difference *^/#^ (*p* < 0.05), **^/##^ (*p* < 0.01), ***^/###^ (*p* < 0.001), and ****^/####^ (*p* < 0.0001), was observed in the % cell viability vs. untreated cells (*) and H_2_O_2_ treated cells (#). Abbreviations: ∆Ψm: mitochondrial membrane potential.

We optimized the concentration of H_2_O_2_ (200 μM) and time of induction (2 h), which resulted in approx. 50% of cell death for the experiment. The H_2_O_2_ treatment decreased ∆Ψm by depolarizing the mitochondrial membrane. SH-SY5Y cells were treated for 12 h with Carom extracts, Carvacrol, and Thymol, after which the extracts were removed and incubated for 2 h with H_2_O_2_ (200 μM). Both the extracts exhibited a significant dose-dependent increase in MMP at 50 μg/mL in Carom-H (86.63 ± 0.28%; # *p* < 0.05) and Carom-EA (83.41 ± 3.95%; # *p* < 0.05). No significant effect was seen at the lower concentrations (1 and 10 μg/mL); however, a statistically non-significant increase (73.57 ± 8.8% in Carom-H; 76.68 ± 8.92% in Carom-EA) in MMP was seen at 25 μg/mL (Figure 9 and Supplementary Figure S6). In the case of pure compounds, statistically significant improvement in MMP was observed at 1 μg/mL, so a concentration lower (0.1 μg/mL) than 1 μg/mL was also studied. Carvacrol and Thymol exhibited a statistically significant dose-dependent increase in MMP, with Thymol displaying a better effect in restoring MMP.

Mitochondrial dysfunction is central to several NDs as it increases ROS production, alters mitochondrial structure and permeability, decreases mitochondrial membrane potential, increases the release of Cytochrome C, increases Caspase (−3 and −9) expression, and finally, inflammation and apoptosis. Hence, protecting mitochondrial function using natural compounds is beneficial in preventing ROS/inflammation generation in NDs. Carvacrol exerted antioxidant and anti-inflammatory action to protect against H_2_O_2_-induced mitochondrial dysfunction in SH-SY5Y cells by modulating heme oxygenase-1/carbon monoxide/nuclear factor kappa B (HO-1/CO/NF-kB) signaling [78]. The heme degradation via the HO-1 enzyme generates CO, which in turn inhibits NF-kB, thus linking mitochondrial dysfunction and inflammation. Previously, the mitochondrial protective action of Carvacrol was observed in Fe^2+^ [70] and Cd^2+^ [79] induced toxicity in SH-SY5Y and PC12 cell lines, respectively. In addition, it also improved the mitochondrial homeostasis in 6-Hydroxydopamine hydrobromide (6-OHDA) - induced rat model and cultured SH-SY5Y cells by preventing superoxide formation [80]. Thymol also revoked mitochondrial dysfunction in myocardial infarction rat models [81] and mercuric chloride toxicity in hepatocellular carcinoma (HepG2) cells [82] through its antioxidant activity. However, detailed studies of Thymol in neuroblastoma cell lines are limited.

## 4. Conclusions

In the present work, different extracts of Carom were prepared, and major bioactive components were identified using GC-MS. Different cell-based and biochemical investigations were performed to assess the neuroprotective activity of the Carom extracts and Carvacrol. Additionally, Thymol (an isomer of Carvacrol) was also examined to study the role of the hydroxyl group in affecting various neuroprotective mechanisms. Oxidative stress, via H_2_O_2_ exposure, was generated in the neuroblastoma cell lines as it is an important generator of ROS. Oxidative stress is one of the key factors responsible for AD pathogenesis, as it accelerates abnormal protein aggregation, neuroinflammation, and neuronal death. Carom extracts provided effective neuroprotection by reducing ROS and restoring MMP in cell-based experiments, which could be attributed to their antioxidant behavior. Additionally, the extracts displayed anti-AChE, anti-oligomerization, and fibrilization activities. Comparing the two pure compounds, both displayed similar effects in Aβ-fibrilization inhibition, an effect on ROS, and neuroprotection. However, Carvacrol was found to be a stronger (~10 times) inhibitor of AChE and inhibited the enzyme through a mixed-type inhibition compared to Thymol (competitive inhibition). These results suggested the role of the para-substituted hydroxy group compared to the meta-substitute in AChE inhibition.

On the other hand, Thymol efficiently inhibited Aβ-oligomerization compared to Carvacrol, indicating the importance of the meta-substituted hydroxyl group. The extracts and pure compounds can be categorized as Class II inhibitors (inhibit both oligomerization and fibrilization), stabilizing Aβ conformations that do not facilitate oligomers or fibril formation. Thus, the present study demonstrates the potential of Carom as a multifunctional curative remedy for AD treatment, but further *in vitro* and *in vivo* experiments are required to elucidate the detailed pathways.

## Figures and Tables

**Table 1:** AChE inhibition kinetic parameters for Carom extracts/bioactives.

	Vmax (μmol/min/mg)	Km (mM)	Inhibition
No Inhibitor	1.096	5.02	
Carom-H (50 μg/mL)	1.110	7.08	
Carom-H (100 μg/mL)	1.021	7.96	Competitive
Carom-EA (50 μg/mL)	1.196	7.72	
Carom-EA (100 μg/mL)	1.214	8.64	Competitive
Carvacrol (50 μg/mL)	1.114	14.76	
Carvacrol (100 μg/mL)	1.174	20.15	Mixed
Thymol (50 μg/mL)	1.117	6.37	
Thymol (100 μg/mL)	1.198	7.33	Competitive

## Data Availability

Data is provided in the Supplementary materials.

## Supplementary Materials

The following supporting information can be downloaded at https://www.mdpi.com/article/10.3390/antiox13010009/s1, Figure S1: Phytoconstituents identified in (A) Carom-H and (B) Carom-EA extracts using gas chromatography-mass spectrometry. Figure S2: Aβ-Oligomerization inhibition in the presence of Carom extracts, Carvacrol, and Thymol. Figure S3: Cytotoxicity assay of Carom extracts, Carvacrol, and Thymol on the SH-SY5Y cells. Figure S4: Neuroprotective effect of Carom extracts, Carvacrol, and Thymol in H_2_O_2_-induced oxidative stress in neuroblastoma SH-SY5Y cells. Figure S5: Effect of Carom extracts, Carvacrol, and Thymol on H_2_O_2_-induced ROS production in SH-SY5Y cells. Figure S6: Mitochondrial membrane potential in SH-SY5Y cells exposed to H_2_O_2_ after pre-treatment with Carom extracts and the pure compounds.
