# Supplementary material for: Trachyspermum ammi Bioactives Promote Neuroprotection by Inhibiting Acetylcholinesterase, Aβ-Oligomerization/Fibrilization, and Mitigating Oxidative Stress In Vitro
Source: Antioxidants (Basel). 2023 Dec 20;13(1):9. doi: 10.3390/antiox13010009 (PMC10812417; doi:10.3390/antiox13010009)

Supplementary Information

Supplementary Figure S1: Phytoconstituents identified in (A) Carom-H and (B) Carom-EA extracts using gas chromatography-mass spectrometry.

A

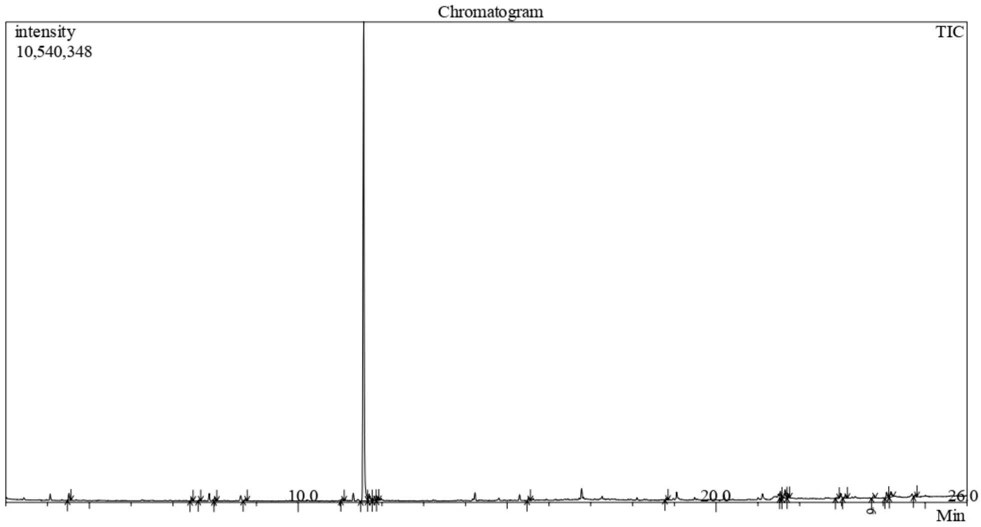

Peak Report TIC

R.Time	Area	Area (%)	Height	Height (%)	Name
4.513	263192	1.11	149690	1.24	Benzene, chloro-
7.445	115900	0.49	70597	0.59	Benzene, 1-methyl-3-(1-methylethyl)-
7.629	41009	0.17	25616	0.21	Benzene, 1,2-dichloro-
8.000	149597	0.63	91071	0.76	.gamma.-Terpinene
8.736	104708	0.44	47184	0.39	5-Isopropyl-2-methylbicyclo[3.1.0]hexan-2-
11.046	82769	0.35	47811	0.40	Thymoquinone
**11.564**	**20539763**	**86.42**	**10504637**	**87.17**	**Phenol, 2-methyl-5-(1-methylethyl)-**
11.697	431002	1.81	145665	1.21	(3-Fluorophenyl)carbamic acid, 2-isopropyl-
11.825	93982	0.40	27016	0.22	2-Isopropyl-5-methyl-1-heptanol
11.909	49560	0.21	29573	0.25	Cyclopropane, 1-(1-hydroxy-1-heptyl)-2-met
15.513	120670	0.51	67246	0.56	2,2,4-Trimethyl-1,3-pentanediol diisobutyrat
18.804	64790	0.27	37489	0.31	3-Benzylsulfonyl-2,6,6-trimethylbicyclo(3.1.
21.536	259757	1.09	149182	1.24	(E)-9-Octadecenoic acid ethyl ester
21.651	408067	1.72	184389	1.53	Vinyltriphenylphosphonium bromide
21.715	78110	0.33	37508	0.31	Phenol, 4,4'-(1-methylethylidene)bis-
22.870	73686	0.31	16741	0.14	2-Oxepanone, 7-hexyl-
23.089	168575	0.71	77350	0.64	2H-Pyran, 2-(2-heptadecynyloxy)tetrahydro-
23.774	138516	0.58	64783	0.54	9-Octadecenoic acid, 1,2,3-propanetriyl ester
24.083	220914	0.93	120209	1.00	1,2-Propanediol, 3-benzyloxy-1,2-diacetyl-
24.179	279529	1.18	114852	0.95	Glycidyl oleate
24.761	83299	0.35	42202	0.35	Triphenylphosphine oxide
	23767395	100.00	12050811	100.00	

B

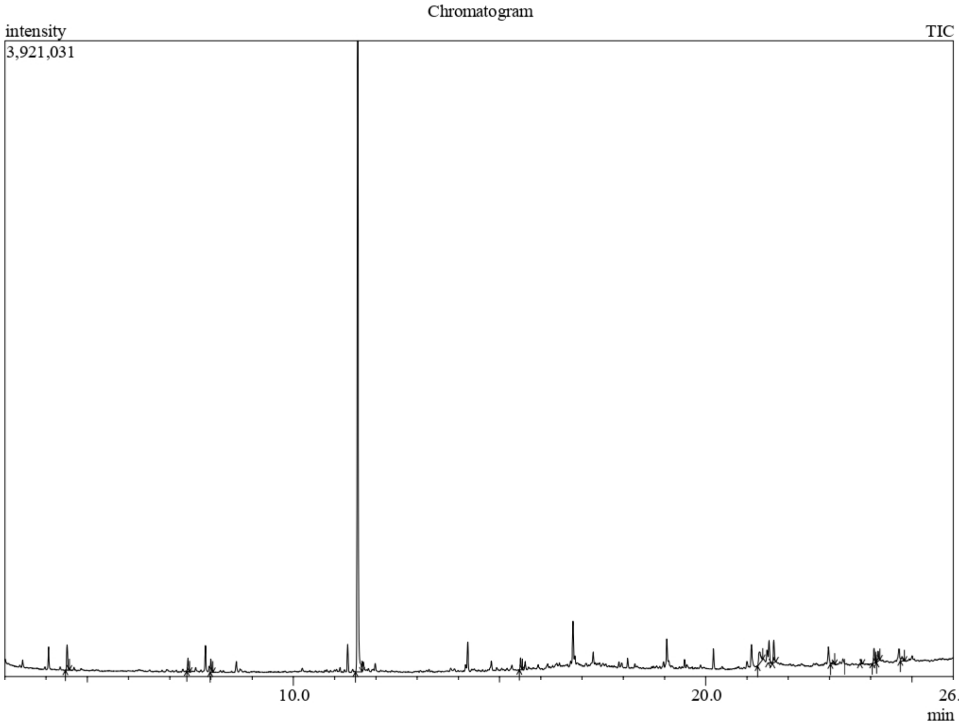

Peak report

R.Time	Area	Area (%)	Height	Height (%)	Name
4.511	268416	3.00	156710	3.23	Benzene, chloro-
7.442	133611	1.49	82239	1.69	Benzene, 1-methyl-3-(1-methylethyl)-
7.998	129334	1.45	79561	1.64	.gamma.-Terpinene
**11.559**	**7060317**	**78.88**	**3892203**	**80.15**	**Phenol, 2-methyl-5-(1-methylethyl)-**
15.511	126344	1.41	73524	1.51	Pentanoic acid, 2,2,4-trimethyl-3-carboxyiso
21.305	274490	3.07	72409	1.49	cis-9-Hexadecenal
21.535	242211	2.71	136823	2.82	cis-9-Octadecenoic acid, propyl ester
21.650	257608	2.88	137987	2.84	Vinyltriphenylphosphonium bromide
23.087	44584	0.50	19728	0.41	2H-Pyran, 2-(2-heptadecynyloxy)tetrahydro-
23.770	45230	0.51	28189	0.58	trans-9-Octadecenoic acid, pentyl ester
24.082	173660	1.94	91336	1.88	1,2-Propanediol, 3-benzyloxy-1,2-diacetyl-
24.179	127538	1.42	61769	1.27	Glycidyl oleate
24.761	67044	0.75	23834	0.49	Triphenylphosphine oxide
	8950387	100.00	4856312	100.00	

Supplementary Figure S2: Aβ-Oligomerization inhibition in the presence of Carom extracts, Carvacrol, and Thymol. The signal intensity of Aβ oligomers at 0, 2, and 4 h. All data are expressed as mean ± SEM (n = 3). A significant difference ** ($p < 0.01$) using two-way ANOVA followed by Dunnett's posthoc was observed in the oligomerization reduction vs. the negative control (Buffer + Aβ).

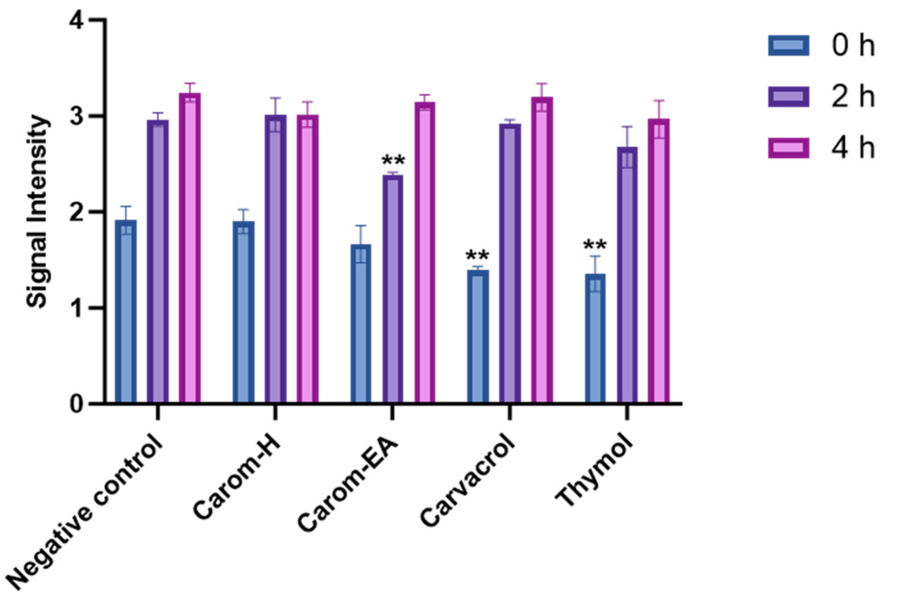

Supplementary Figure S3: Cytotoxicity assay of Carom extracts, Carvacrol, and Thymol on the SH-SY5Y cells. The cells were treated with varying concentrations (1, 10, 25, and 50 μg/ml) of extract/bioactive for 24 h. The absorbance values (Y-axis) were plotted against concentration (X-axis). The data were expressed as mean ± SD (*n* = 3). A significant difference * ($p < 0.05$) was observed using one-way ANOVA followed by Dunnett's post-hoc.

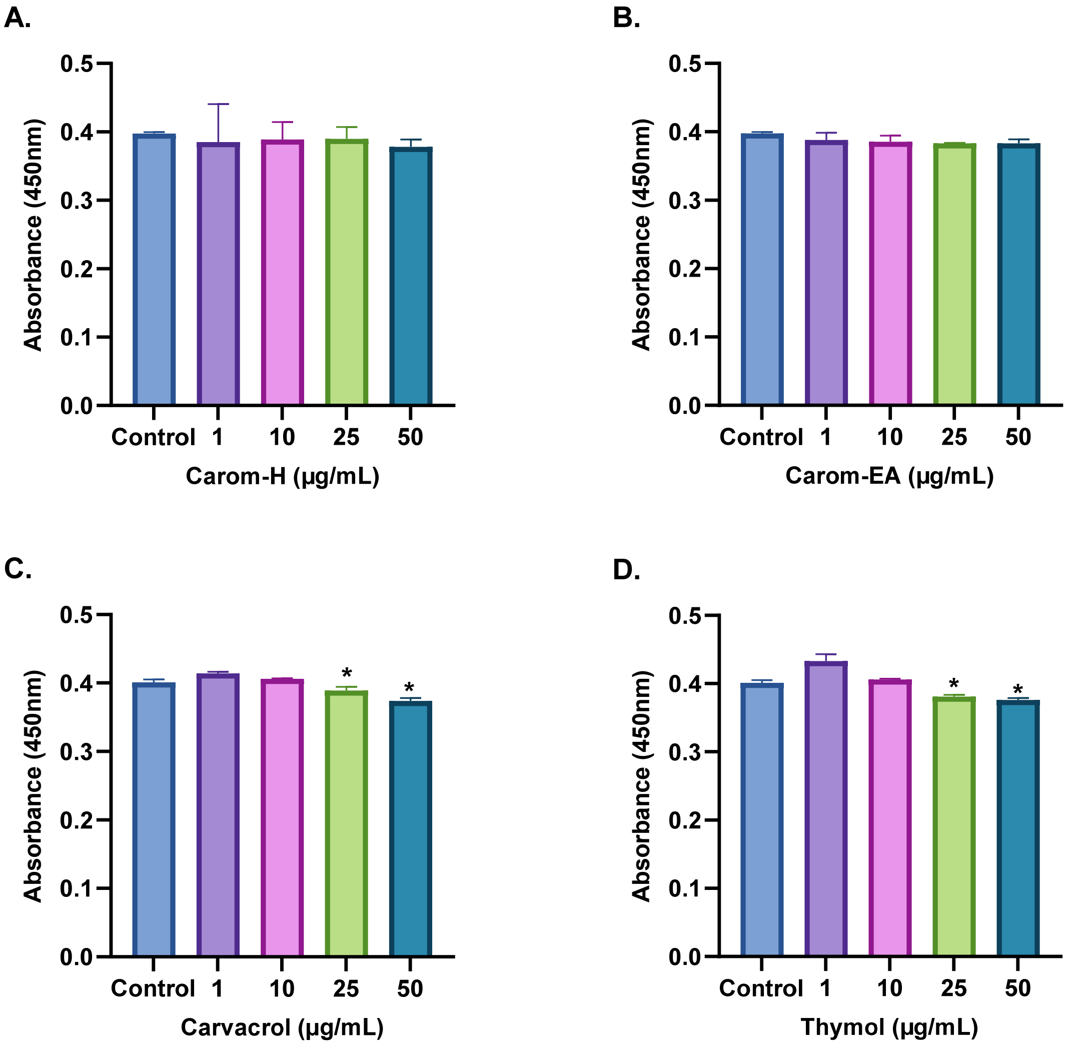

Supplementary Figure S4: Neuroprotective effect of Carom extracts, Carvacrol, and Thymol in H_2O_2-induced oxidative stress in neuroblastoma SH-SY5Y cells. The SH-SY5Y cells were pre-treated with various concentrations of the extract/bioactives (0.1, 1, 10, and 50 μg/ml) for 12 h followed by 6 h of H_2O_2 (100 μM) treatment. The absorbance values (Y-axis) were plotted against concentration (X-axis). The data were expressed as mean ± SD (n = 3). A significant difference $^{*/\#}$ ($p < 0.05$), $^{**/\#\#}$ ($p < 0.01$), and $^{****/\#\#\#\#}$ ($p < 0.0001$) using one-way ANOVA followed by Dunnett's test was observed in the treated vs untreated (control) cells (*) and H_2O_2 treated cells (#).

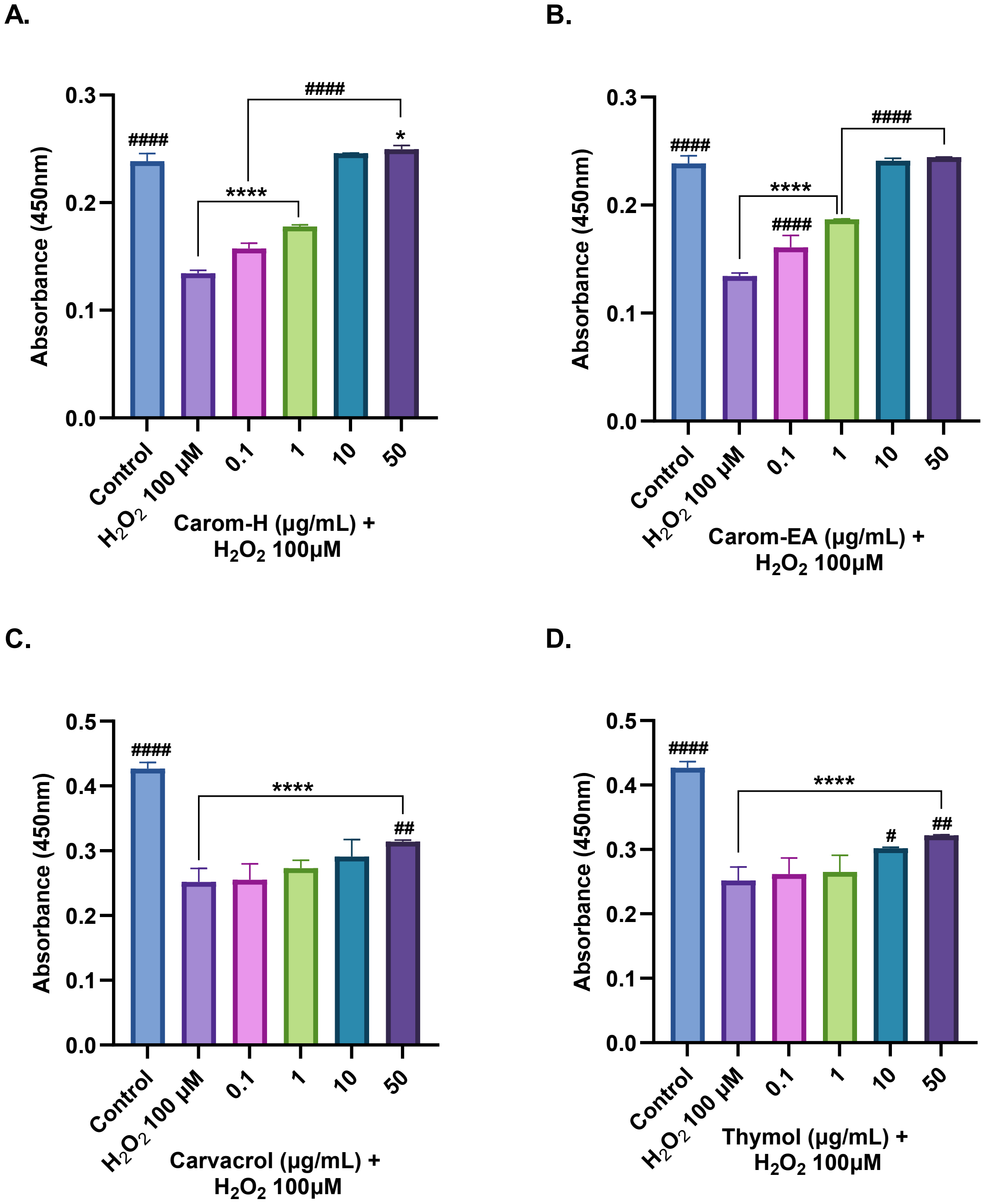

Supplementary Figure S5: Effect of Carom extracts, Carvacrol, and Thymol on H_2O_2-induced ROS production in SH-SY5Y cells. The SH-SY5Y cells were pre-incubated for 12 h with varying concentrations (1, 10, 25, and 50 µg/ml) of the extracts/bioactives followed by 4h H_2O_2 (100 µM) exposure. The fluorescence values (Y-axis) were plotted against concentration (X-axis). The data were expressed as mean $\pm$ SD ($n = 3$). The data analysis was performed by One-way ANOVA followed by Dunnett's test. A significant difference $^{*/\#}$ ($p < 0.05$), $^{**/\#\#}$ ($p < 0.01$), $^{***/\#\#\#}$ ($p < 0.001$), and $^{****/\#\#\#\#}$ ($p < 0.0001$) was observed in the treated vs untreated (control) cells (*) and H_2O_2 treated cells (#).

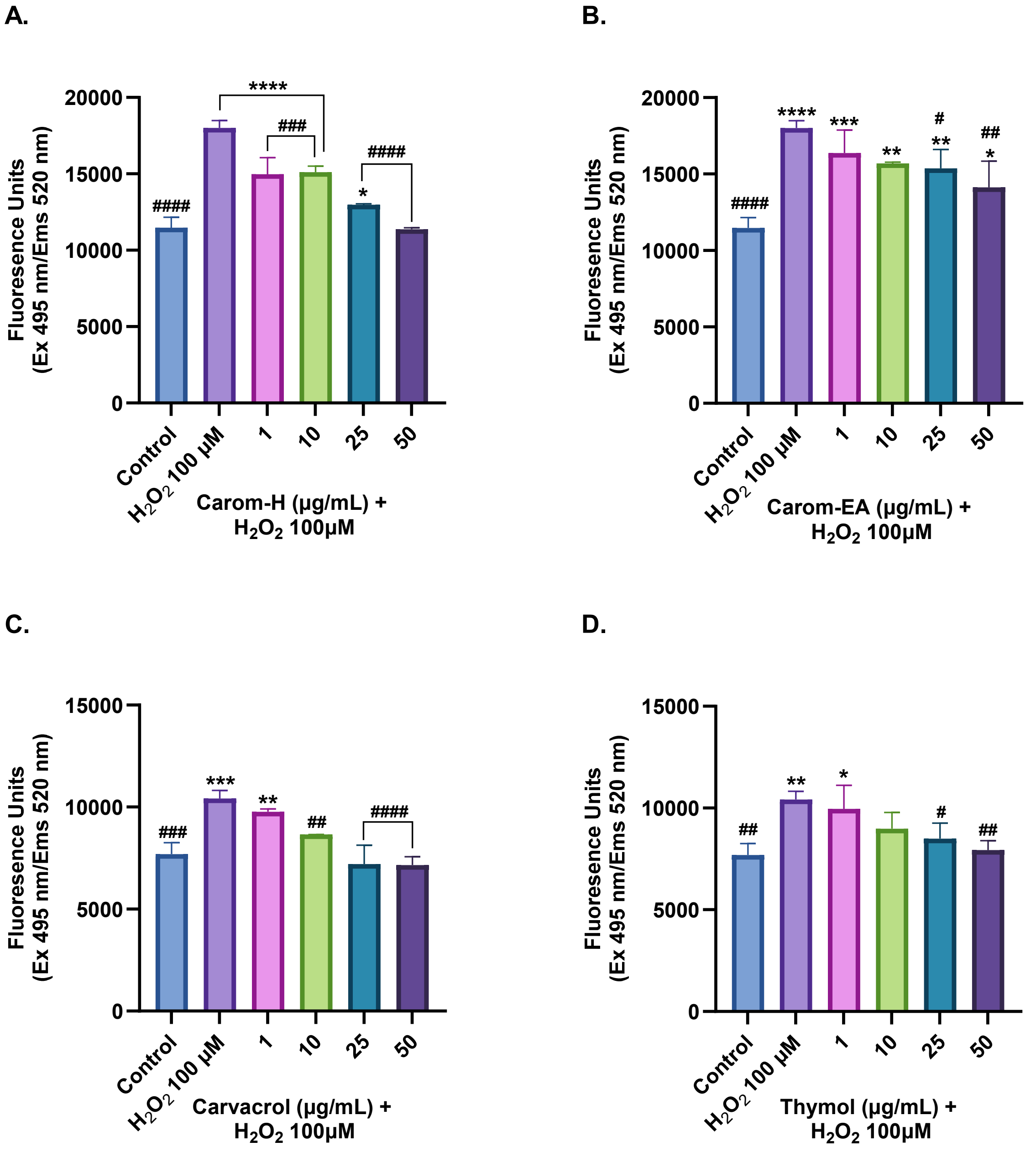

Supplementary Figure S6: Mitochondrial membrane potential in SH-SY5Y cells exposed to 200 μM H_2O_2 for 2 h after 12 h pre-treatment with Carom extracts (1, 10, 25, and 50 μg/ml) and the pure compounds (0.1, 1, 10 and 25 μg/ml). The results were expressed as mean ± SD (*n* = 3). The fluorescence values (Y-axis) were plotted against concentration (X-axis). The data analysis was performed by One-way ANOVA followed by Dunnett's test. A significant difference $^{*/\#}$ ($p < 0.05$), $^{**/\#\#}$ ($p < 0.01$), $^{***/\#\#\#}$ ($p < 0.001$), and $^{****/\#\#\#\#}$ ($p < 0.0001$), was observed in the treated vs untreated (control) cells (*) and H_2O_2 treated cells (#). Abbreviation: ΔΨm: Mitochondrial membrane potential.

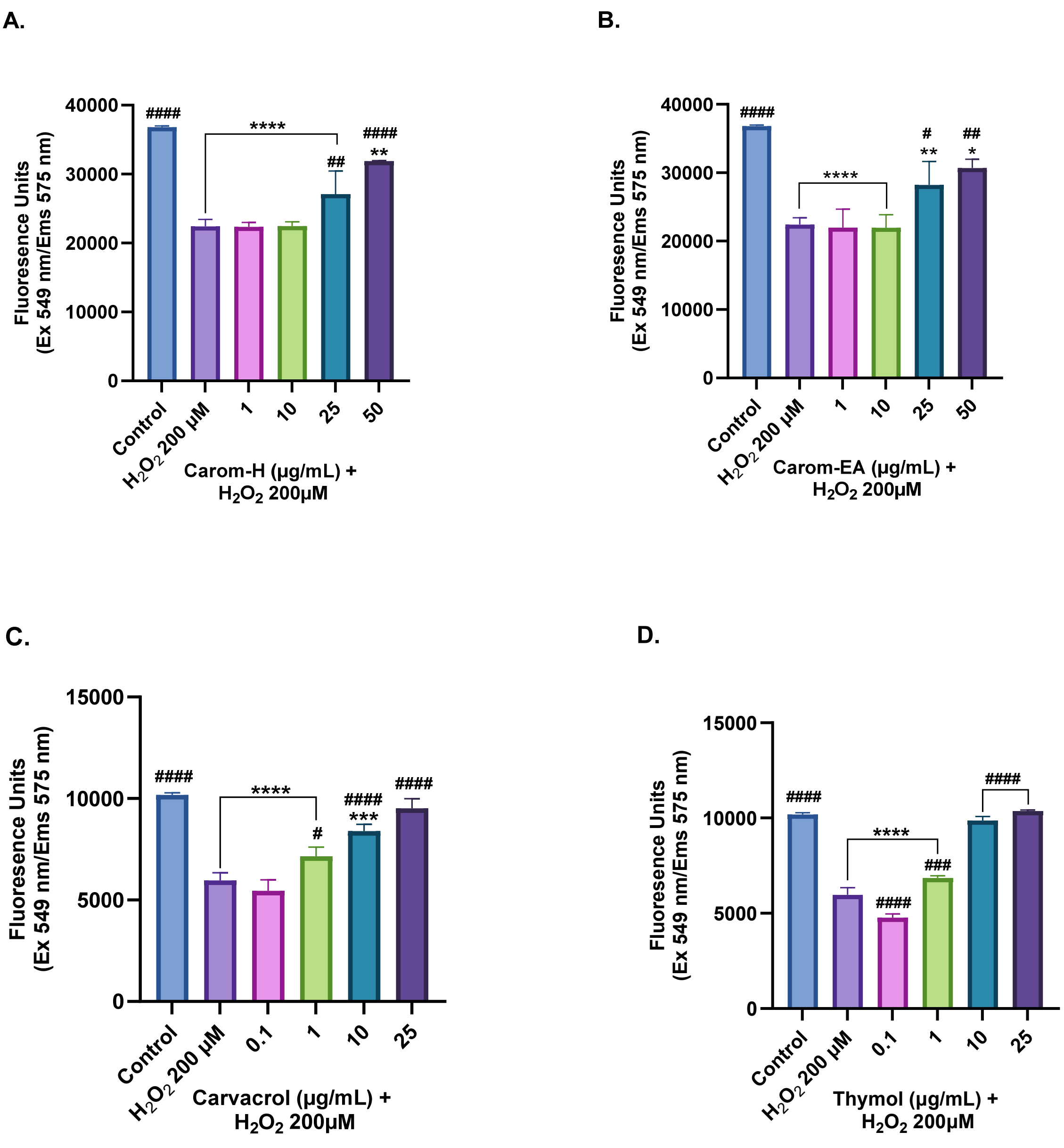

Supplement: Supplementary file 1 [file antioxidants-13-00009-s001.zip › antioxidants-2732489-supplementary.pdf]
